# Selenium-Catalyzed Reduction of Hydroperoxides in Chemistry and Biology

**DOI:** 10.3390/antiox10101560

**Published:** 2021-09-30

**Authors:** Laura Orian, Leopold Flohé

**Affiliations:** 1 Dipartimento di Scienze Chimiche, Università degli Studi di Padova, 35131 Padova, Italy; 2 Dipartimento di Medicina Molecolare, Università degli Studi di Padova, 35121 Padova, Italy; 3 Departamento de Bioquimica, Universidad de la Republica, Montevideo 11800, Uruguay

**Keywords:** catalysis, deselenylation, diphenyl diselenide, ebselen, glutathione peroxidases, hydrogen peroxide, hydroxy perhydroxy selenane, selenocysteine, selenenic acid

## Abstract

Among the chalcogens, selenium is the key element for catalyzed H_2_O_2_ reduction. In organic synthesis, catalytic amounts of organo mono- and di-selenides are largely used in different classes of oxidations, in which H_2_O_2_ alone is poorly efficient. Biological hydroperoxide metabolism is dominated by peroxidases and thioredoxin reductases, which balance hydroperoxide challenge and contribute to redox regulation. When their selenocysteine is replaced by cysteine, the cellular antioxidant defense system is impaired. Finally, classes of organoselenides have been synthesized with the aim of mimicking the biological strategy of glutathione peroxidases, but their therapeutic application has so far been limited. Moreover, their therapeutic use may be doubted, because H_2_O_2_ is not only toxic but also serves as an important messenger. Therefore, over-optimization of H_2_O_2_ reduction may lead to unexpected disturbances of metabolic regulation. Common to all these systems is the nucleophilic attack of selenium to one oxygen of the peroxide bond promoting its disruption. In this contribution, we revisit selected examples from chemistry and biology, and, by using results from accurate quantum mechanical modelling, we provide an accurate unified picture of selenium’s capacity of reducing hydroperoxides. There is clear evidence that the selenoenzymes remain superior in terms of catalytic efficiency.

## 1. H_2_O_2_ Reduction by Organoselenides

In recent years, the success of H_2_O_2_ as oxidizing agent has been increasing. Hydrogen peroxide is the ideal oxidant because it is powerful and eco-friendly. Its strength directly descends from the weakness of the oxygen-oxygen bond, whose disruption ultimately leads to water as the only product. This makes the use of H_2_O_2_ attractive, especially because it is less dangerous than oxygen/organic mixtures and more stable overall than many organic peroxides. For large scale production, also the relatively low cost of H_2_O_2_ is advantageous. Its strongest limitation is perhaps the presence of water as solvent in the commercial H_2_O_2_, which unavoidably makes oxidations slow. Oxidations by H_2_O_2_ benefit from the presence of a catalyst and for this purpose organoselenides have proved particularly convenient. The combination of selenium and hydrogen peroxide is an efficient approach to different important classes of organic reactions, to which Bayer-Villiger oxidations of carbonylated substrates, i.e., aldehydes and ketones, oxidations of alcohols and nitrogen containing compounds and alkene epoxidations belong [1,2,3,4,5,6,7,8]. In the last decade, examples of this catalysis have been reported also in water, paving the route to the use of selenium in green chemistry and moving further apart from the use of toxic heavy metal compounds, like dichromates and permanganates [9,10].

**Scheme 1:**
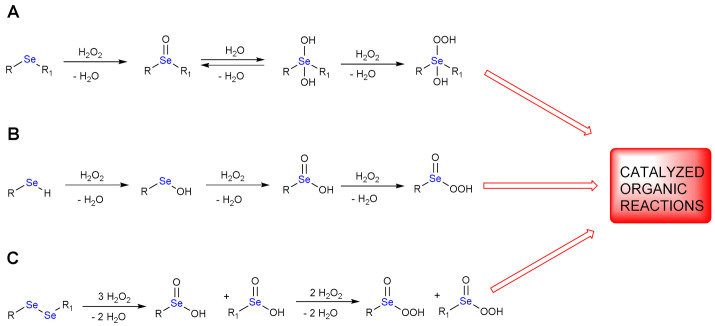
Oxidations of mono– and di–organoselenides by H_2_O_2_; the catalytic starting material may be a dialkylselenide or diarylselenide (**A**), a selenol (**B**) or a diselenide (**C**).

A catalytic amount of organoselenides can reduce one or more equivalents of H_2_O_2_, while selenium is concomitantly oxidized. In fact, the oxidation state of this chalcogen can progressively increase from −2 to +6. The perseleninic acid, in which selenium oxidation state is +4, has been proposed as the real oxidizing species, active in place of H_2_O_2_. Thus, selenium in organic synthesis behaves like an effective oxygen transfer agent. The oxidation mechanism of mono- and di-organoselenides is shown in Scheme 1.

The oxidation of a monoselenide (RSeR_1_) gives a single stable product, i.e., a selenoxide (Scheme 1A, first elementary step). If we consider a selenol (RSeH, R_1_ = H, Scheme 1B), subsequent oxidations occur leading to selenenic, seleninic and perseleninic acid/selenonic acid, respectively (Scheme 1B). Recently, Back and coworkers have reported evidence of formation of benzene peroxyselenonic acid in the epoxidation of cyclooctene promoted by selenonic acid/H_2_O_2_ [11].

The oxidation of a diselenide is more complex because the cleavage of Se-Se bond and the formation of seleninic products occurs after the addition of three equivalents of H_2_O_2_ (Scheme 1C, first reaction). The seleninic products can be further oxidized to perseleninic derivatives (Scheme 1C, second reaction). 

The oxidation path of diselenides by H_2_O_2_ or generally by peroxides was first proposed by Kice and Chiou [12], who described the formation of selenoxide and reduced species such water or alcohol molecules. As example, we can consider diphenyl diselenide (**1**), which is the most used catalyst in organic synthesis, but has also been thoroughly studied as antioxidant drug [13].

**Scheme 2:**
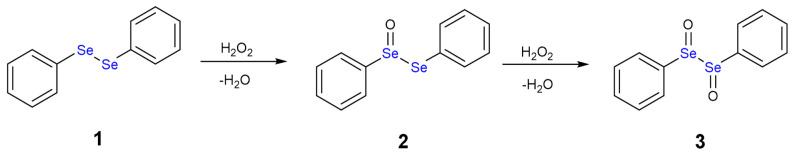
Oxidation of diphenyl diselenide (**1**) by H_2_O_2_.

**Scheme 3:**
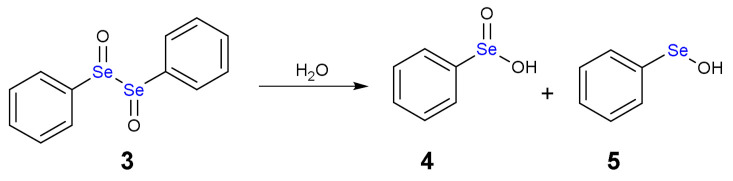
Hydrolysis of diphenyl diselenoxide (**3**).

The addition of one equivalent of H_2_O_2_ leads to the formation of a selenoxide (**2**). Notably, selenium is a stereogenic center and potentially two diastereoisomers with identical energy form. The addition of a second equivalent of H_2_O_2_ may oxidize also the adjacent Se atom with the formation of a diselenoxide (**3**) (Scheme 2), which is not stable and the selenium-selenium bond breaks. In fact, experimentally, the formation of benzene seleninic acid (**4**) has been detected. Thus, at a certain stage the inter-chalcogen bond of the diselenoxide may be hydrolized to benzene seleninic (**4**) and benzene selenenic (**5**) acids (Scheme 3).

This latter product, like all selenenic derivatives, is experimentally elusive and has not been detected. Only when the selenium is protected by cradle-shaped molecular structure, selenenic acids could be synthetized [14,15].

**Scheme 4:**
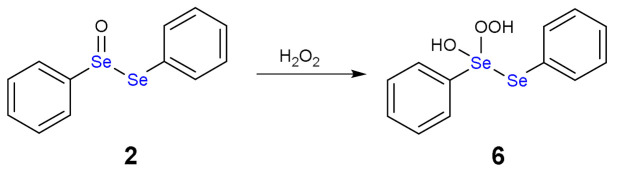
Formation of hydroxy perhydroxy selenane (**6**).

**Scheme 5:**
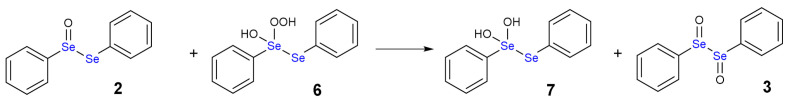
Oxidation of diphenyl selenoxide (**2**) by hydroxy perhydroxy selenane (**6**).

As alternative, both H_2_O_2_ molecules can attack the same Se atom forming a hydroxy perhydroxy species (**6**) (Scheme 4). This intermediate was never detected either, although a nice study by Braga and co-workers postulated its formation [16]. Then, the hydroxy perhydroxy intermediate can oxidize the selenoxide to diselenoxide, according to the mechanism described in Scheme 5.

**Scheme 6:**
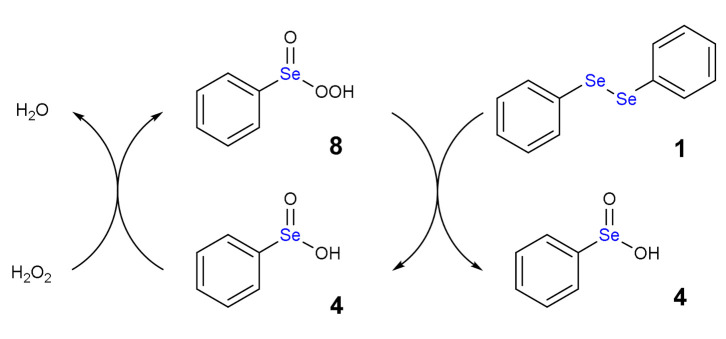
Autocatalytic mechanism of the oxidation by diphenyl diselenide by H_2_O_2_: **8** is reduced to **4** with concomitant oxidation of **1**.

Another intriguing aspect is that the oxidation process of diphenyl diselenide is autocatalytic, as explained in the mechanism sketched in Scheme 6 [17]. 

Using an excess of H_2_O_2_, the benzene perseleninic acid (**8**, Scheme 6), which is a powerful oxidant, forms. This product is the real oxidizing agent in organic reactions, when H_2_O_2_ is used in presence of a catalytic amount of diphenyl diselenide (Scheme 1).

Theoretical studies have played an important role in understanding the intimate mechanistic features of the oxidation of organoselenides by H_2_O_2_. Nowadays, thanks to the supercomputing facilities, quantum mechanical (QM) calculations, particularly Density Functional Theory (DFT) calculations, provide an accurate description of the potential energy surface (PES). The reactions occur through the location of the minimum energy points, corresponding to the reactants and products and, more in general, to the intermediates, as well as of the transition states. The latter connect reactants and products, and determine the energy barrier of each elementary step. This means that a mechanistic path can be explored entirely in silico by computing the equilibrium structures of all the involved species and their energies, as well as the structure and energy of transition states once they have been correctly identified. Particularly, the information on the energetics of different plausible paths is precious to identify those that are thermodynamically as well as kinetically preferred. It is worth to remember that quantum mechanical calculations are carried out considering a single molecule, while thermodynamics and kinetic quantities are related to the Gibbs free energies, which are collective properties of the chemical system. Thus, corrections can be added to the energies using e.g., statistical gas equations. In addition, the effects of solvation can also be added as a correction to the energies, employing the so-called dielectric continuum models, like PCM (Polarizable Continuum Model) [18] and COSMO (Conductor-like Screening Model for solvation) [19]. Notably, insightful results are obtained using DFT mechanistic approaches and the exploration of the PES provides information on the chemical reactivity, which, combined with the experiment, can elucidate fundamental atomistic details. The limitation of these studies is due to the size of the system, which typically is formed by no more than 100–150 atoms, but computational protocols based on an intelligent choice of the system itself can be efficiently used to study reactivity even in the biological environment, like in enzymatic pockets. 

After more than 200 years since the discovery of selenium by Berzelius [20], the role of this chalcogen in biology is still under debate. A fundamental, albeit yet not complete, justification is certainly rooted in the chemical properties of this element, particularly in those displayed in the elementary reactions involved in the biological phenomena. To this purpose, since selenium is found in proteins mainly designated to balance hydroperoxide challenge, metabolic regulation and signalling, a systematic theoretical investigation of H_2_O_2_ reduction potential of this chalcogen, extended to its siblings sulfur and tellurium, has been carried out in the last decade. 

Several authors tackled the problem of benchmarking the best performant approach for describing the chemistry of organochalcogenides. In 2010, Heverly-Coulson and Boyd [21] tested several combinations of functionals/basis sets and concluded that an optimal approach is B3PW91 exchange-correlation (XC) functional combined with Pople double/triple ζ Gaussian type orbital (GTO) basis set, like 6-31G(d,p) or 6-311+G(2df,p). Zaccaria et al. [22] performed a DFT benchmark focusing on diaryl dichalcogenides, testing twenty-three density functionals in combination with Slater type orbital (STO) basis sets of increasing size (TZP, TZ2P, QZ4P); in this study, relativistic effects were considered using scalar ZORA approximation. The major outcomes are that GGA like OPBE or dispersion corrected such as BP86-D3(BJ) and BLYP-D3(BJ) functionals, in combination with TZ2P-sc (small frozen core) are recommended to obtain reliable geometries, while ZORA-OLYP/TZ2P-sc represents an optimal choice to predict the energetics, since a correct decreasing bond strength from Se-Se to Te-Te has been found [22]. For the sake of clarity, the acronyms here used are explained in the Abbreviations at the end of the text.

We here describe some model organoselenides, whose oxidation mechanism by H_2_O_2_ has been accurately studied. A rather exhaustive and recent overview including additional experimental and theoretical mechanistic studies can be found in Ref. [23] dedicated to monoselenides and in Ref. [24] dedicated to diselenides. The mechanism of oxidation of an organoselenide and more in general of an organochalcogenide by H_2_O_2_ consists in a nucleophilic attack of the chalcogen (S, Se, Te) to one oxygen of the peroxide with concomitant transfer of its proton to the second oxygen, O-O bond breaking and formation of a water molecule. Like all S_N_2 reactions, in gas phase, the reactants, i.e., the nucleophile and the susbtrate, approach and form the so-called reactant complex (RC), which is stabilized with respect to the free reactants. The reaction proceeds crossing the transition state (TS) until a product complex (PC) forms, which is also stabilized with respect to the free products. The nucleophilic attack of an organochalcogenide to H_2_O_2_ is in general characterized by a double-well energy profile in gas phase, which is recovered also in condensed phase. Notably, other S_N_2 involving organochalcogenides display single or triple-well profiles, depending on the nature of the nucleophile, of the substrate and of the medium [25]. 

In the following, (i) the reactivity will be quantitatively described and interpreted using electronic energies and (ii) the energy barriers are computed taking as reference the free reactants, unless differently specified.

### 1.1. Oxidation of n-butyl Phenyl Selenide by H_2_O_2_

**Scheme 7:**
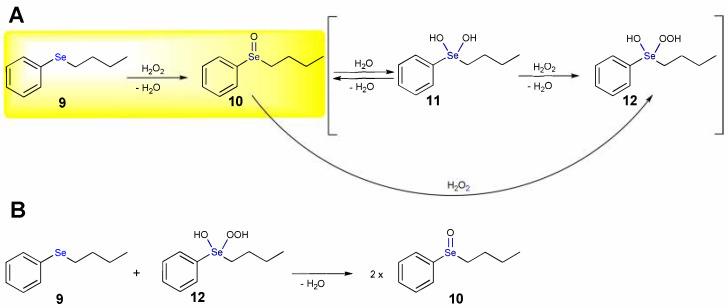
Oxidation mechanism of n-butyl phenyl selenide by H_2_O_2_ (**A**) and by the hydroxy perhydroxy selenane **12** (**B**). The direct oxidation step is highlighted.

The oxidation of n-butyl phenyl selenide (**9**) by H_2_O_2_ leads to the corresponding selenoxide labelled as **10** (highlighted step in Scheme 7A). At ZORA-OPBE/TZ2P level of theory (see Abbreviations), a barrier for the direct oxidation of 16.2 kcal mol^−1^ is computed and the product is stabilized by 36.8 kcal mol^−1^ [17]. By addition of a water molecule, the dihydroxy derivative **11** may form with an activation energy of 22.8 kcal mol^−1^, which is destabilized by 13.1 kcal mol^−1^ (Scheme 7A). In presence of H_2_O_2_, **11** can be converted into the hydroxy perhydroxy selenane **12** (Scheme 7A), which is further destabilized by 3.3 kcal mol^−1^; this step has an energy barrier of 15.6 kcal mol^−1^ [17].

The direct conversion of **10** to **12** (Scheme 7A) has an activation energy of 15.0 kcal mol^−1^, suggesting that it likely occurs in one step, rather than passing through **11**. The hydroxy perhydroxy selenane **12** is considered the active oxidizing agent in selenium catalyzed organic reactions. Based on the calculations, the possibility that **12** reacts with **9** (Scheme 7B) is excluded, because this process has an activation energy of 23.6 kcal mol^−1^. This outcome is in nice agreement with the lack of evidence for an autocatalytic mechanism in the kinetic profile [17].

### 1.2. Oxidation of Phenylselenol by H_2_O_2_

**Scheme 8:**
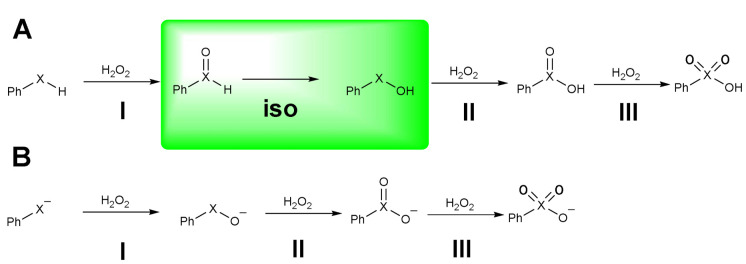
Oxidation of phenylchalcogenols in neutral (**A**) and anionic form (**B**) (X = S, Se, Te). The isomerization step is highlighted.

Very recently, the oxidation of model phenylchalcogenols PhXH (X = S, Se, Te) has been investigated in silico, spanning all the relevant oxidation states, with the aim of assessing the different redox behavior of the three chalcogens [26]. Phenylselenol was chosen because it is a precursor of the benzene peroxyseleninic acid, which is the real oxidizing agent in the oxidations catalyzed by diphenyl diselenide (see Section 1.3), and because arylselenols are important targets for chemoprevention for the low toxicity of their metabolites. The calculations were carried out at ZORA-OLYP/TZ2P level of theory, i.e., the optimal approach benchmarked for these reactions involving chalcogens. Both the neutral and the anionic form were considered and sequential reactions with up to three equivalents of H_2_O_2_ were described (Scheme 8) [26].

The main difference is due to the isomerization step (**iso**) which occurs after the first oxidation (step **I**, Scheme 8A) but only when starting from the neutral phenylselenol, the case which will be discussed first. The activation energies are shown in Table 1.

**Scheme 9:**
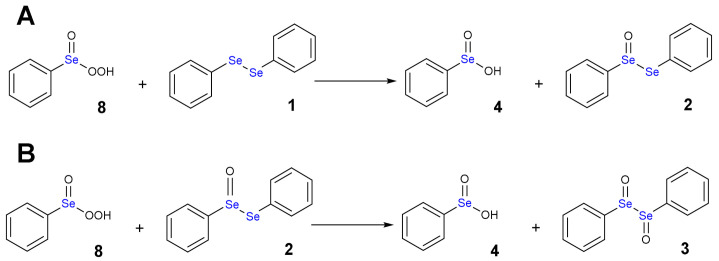
Oxidation of diphenyl diselenide **1** (**A**) and diphenyl selenoxide **2** (**B**) by perseleninic acid **8**.

The barriers have not a monotonic trend when increasing the oxidation state of the chalcogen. Apart from the step **iso**, which is characterized by a rather high barrier even for Se and Te, a significant decrease is observed when going from the first to the second oxidation, i.e., from **I** to **II**, and the trend S > Se > Te is maintained (Table 1). Surprisingly, in the third oxidation (**III**), an inversion of the trend is observed, and it is predicted that Se has the highest barrier (Table 1). This last step, in which the chalcogen has lost nucleophilic power because of its oxidation state, has a neatly different barrier trend, i.e., Se > Te > S (Table 1), accounting for the tendency of sulfur derivatives to reach the highest oxidation state. In the study by Bortoli et al. [26], this has been explained observing that, due to the nature of these reactions, the energy of the HOMOs of the phenylchalcogenols are related to the energies required by the oxidations. Particularly, the lower the HOMO of the reactant, the higher the barrier. This must be ascribed to a less favorable interaction with the empty orbitals of H_2_O_2_. Indeed, the benzene seleninic acid has the lowest HOMO, lower than benzene sulfinic and benzene tellurinic acids, fully justifying the highest barrier for the oxidation to benzene selenonic acid (step **III**). This outcome has impact on the biological role of selenium, whose chemical nature prevents overoxidation, differently from sulfur. This characteristic behaviour is observed also when considering the sequential oxidations of phenylchalcogenolates, (Scheme 9B) whose activation energies are shown in Table 2 [26].

The energy values change significantly in presence of the negative charge since the nucleophilicity of the anionic chalcogen is increased. Importantly, both in gas-phase and in water, the behavior of the heavier selenium and tellurium is strongly different from sulfur: their oxidation is initially easier than sulfur, but becomes more and more difficult with increasing oxidation state, while the opposite is found for sulfur (Table 2). Also the results of this accurate computational analysis suggest that the chemical nature of selenium prevents the overoxidation, which, in biology, would irreversibly impair the enzymatic activity. Furthermore, this supports the hypothesis of several authors who establish the peculiar biological role of selenium also in its redox properties [27]. 

### 1.3. Oxidation of Diphenyl Diselenide by H_2_O_2_

The oxidation of diphenyl diselenide **1** by H_2_O_2_ has a quite complex mechanistic path, which has been thoroughly investigated by Ribaudo et al. [17] in a combined NMR and computational study. At ZORA-OPBE/TZ2P level of theory, the oxidation of **1** takes to the formation of the selenoxide **2** through a reaction barrier of +19.1 kcal mol^−1^ and with a reaction energy of −38.1 kcal mol^−1^. Then, the doubly oxidized product **3** may form upon the addition of a second equivalent of H_2_O_2_ with similar activation energy [17]. Particularly, due to the stereogenic nature of the chalcogens, different energetics have been reported for the formation of (R,R) and (R,S) diselenoxide, i.e., activation energies of +18.9 and +19.4 kcal mol^−1^ and reaction energies of −38.3 and −35.3 kcal mol^−1^, respectively [17]. In the attempt of explaining the sigmoidal shape of the kinetics, which indicates that the process is autocatalytic, the hypothesis sketched in Scheme 5 was initially explored. The addition of the second equivalent of H_2_O_2_ to the selenoxide **2** does not lead to the formation of the diselenoxide **3**, but the hydroxy perhydroxy selenane **6** may form (the barrier is +14.7 kcal mol^−1^ and the product is destabilized by +12.5 kcal mol^−1^). The latter may react with the diphenyl diselenide **1** or with the selenoxide **2**, with an activation energy in both cases close to 20.0 kcal mol^−1^ and a large negative reaction energy, i.e., close to −50.0 kcal mol^−1^. The products are the selenoxide **2** and water and the selenoxide **2**, the diselenoxide **3** and water, respectively [17]. Also, the diselenoxide **3** can be transformed into a hydroxy perhydroxy selenane by addition of H_2_O_2_ (barrier +19.1 kcal mol^−1^ and destabilized by +8.3 kcal mol^−1^) and it can react with either **1** or more likely with **2**, in analogous fashion to what is shown for the monooxidized selenoxide **2** (Scheme 5). For the latter reaction, a rather large negative reaction energy of −46.6 kcal mol^−1^ is computed [17]. Based on the energetics, it emerges that the mechanism sketched in Scheme 2 is the most plausible (‘direct oxidation’), and the autocatalytic mechanism should start once the Se-Se bond is cleaved, a process which is easier for the diselenoxide **3** (Scheme 3) [17]. Both monoselenides, i.e., the benzene selenenic acid **5** and the benzene seleninic acid **4**, can be oxidized to perseleninic acid **8**. The former requires one equivalent of H_2_O_2_ to be oxidized to **4** (activation energy of +12.6 and reaction energy of −49.2 kcal mol^−1^), which can be converted into the perseleninic acid **8** by addition of a second equivalent of H_2_O_2_ (activation energy of +24.8 and reaction energy of −5.5 kcal mol^−1^). At this point, the autocatalytic mechanism is triggered, as sketched in Scheme 6 [17]. The perseleninic acid **8** can oxidize the diphenyl diselenide **1** (activation energy of +11.8 and reaction energy of −32.6 kcal mol^−1^) as well as the diphenyl selenoxide **2** (activation energy of +11.4 and reaction energy of −32.8 kcal mol^−1^); these reactions and their products are explicitly shown in Scheme 9A,B, respectively. 

## 2. Oxidation of Cysteine and Selenocysteine by H_2_O_2_

In the last two decades, the mechanism of oxidation of cysteine (Cys) and selenocysteine (Sec) by H_2_O_2_ has been investigated in silico by several authors using different approaches. These theoretical studies are important, because they represent the simplest attempt to link the redox chemistry of thiols/selenols to the sulfur/selenium activity in the peroxidatic proteins. Particularly, referring to GPx family, the activity of the sulfur mutant of GPx (S-GPx) is orders of magnitude lower than the wild-type selenoenzyme [28,29]. In addition, the rate constant of H_2_O_2_ reduction by free deprotonated cysteine is six orders of magnitude smaller than that reported for S-GPx [30,31,32,33]. So it is clear that we cannot ascribe the differences of GPx enzymatic reactivity merely to the different acidity of the couples selenol/selenolate and thiol/thiolate, whose p*K*_a_ is 5.2 [34] and 8.4, respectively [35]. These aspects were elucidated by Enescu and Cardey focussing on the oxidation by hydrogen peroxide of the model compounds methylthiolate/selenolate [36] and deprotonated Cys/Sec employing accurate QCISD(T) and MPn calculations in gas as well as in condensed phase [37]. In the former case, no important differences are predicted between the thiolate and selenolate kinetics; the calculated activation Gibbs free energies are 20.7 and 19.2 kcal mol^−1^ (in gas-phase) and 18.4 and 16.5 kcal mol^−1^ (in water), respectively. Unluckily, these results are not due to the oversimplified models used by the authors. In fact, when moving to the oxidation of the deprotonated Cys/Sec by H_2_O_2_, analogous results are computed. The authors also stress the importance of intramolecular hydrogen bonding leading at different conformers of Cys/Sec, for which the calculated Gibbs free activation energies are significantly different in gas phase as well as in water. Nevertheless, when comparing these energy values using the same conformers for Cys and Sec, it emerges that the energy barriers of the oxidation of free Cys and Sec by H_2_O_2_ are very similar, while in general the barrier height depends on the medium in which the reaction occurs and on the conformation of the residues. These two factors may be controlled in the enzymatic environment, but they are extremely variable when the process occurs in solution. 

Very recently, the mechanistic details of Cys and Sec oxidation by H_2_O_2_ have been systematically studied at ZORA-BLYP-D3(BJ)/TZ2P following three hypotheses: (i) reaction between the peroxide and the neutral residues; (ii) reaction between the peroxide and the neutral residues mediated by few water molecules; (iii) reaction between the peroxide and the anionic residues [38]. The analyses (i) and (ii) were extended also to tellurocysteine (Tec) for completeness; conversely, analysis (iii) was limited to the natural amino acids Cys and Sec and is analogous to the model by Enescu and Cardey described above.

**Scheme 10:**
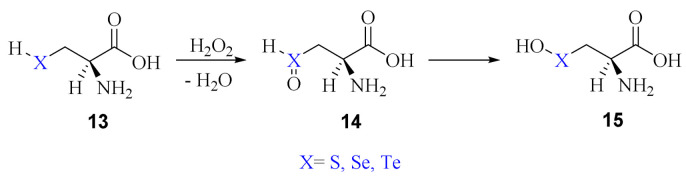
Oxidation of Cys/Sec/Tec by H_2_O_2_ via the initial formation of a chalcogenoxide followed by isomerization to chalcogenenic acid.

The first mechanism involves two steps, i.e., the oxidation of the chalcogenol to chalcogenoxide and the isomerization to the corresponding chalcogenenic acids (Scheme 10).

As for phenylchalcogenol, the isomerization step has a rather high activation energy, which decreases when going from S to Se and Te. Curiously, this mechanism has been found suitable to describe the oxidation of a designed tellurium mutant of GPx (Te-GPx) [39].

**Scheme 11:**
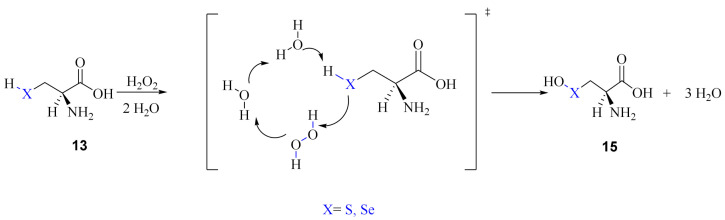
Water assisted oxidation of Cys/Sec by H_2_O_2_ to chalcogenenic acid.

The second mechanism proposed by Madabeni et al. [38] involves few water molecules as required in the so-called solvent-assisted proton-exchange (SAPE) approach. This method has been largely applied to organochalcogen reactivity by Bayse et al. [40,41,42]. Along this path, a proton is transferred through a network of hydrogen bonded water molecules and H_2_O_2_; the reduction of the peroxide occurs in a concerted manner. Particularly, two water molecules were included in the model, as shown in Scheme 11.

**Scheme 12:**
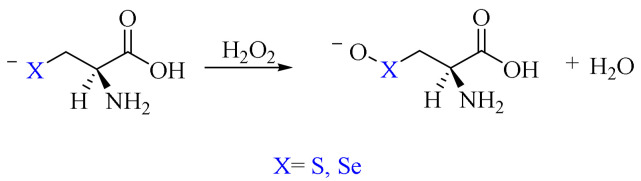
Cys^−^/Sec^−^ oxidation by H_2_O_2_ to deprotonated chalcogenenic acid.

Finally, the direct oxidation of the anionic forms of the residues, i.e., Cys^−^ and Sec^−^, has been considered. The concerted mechanism was previously reported by other authors for these amino acids as well as for small model chalcogenolates (Scheme 12) [36,37].

**Figure 1:**
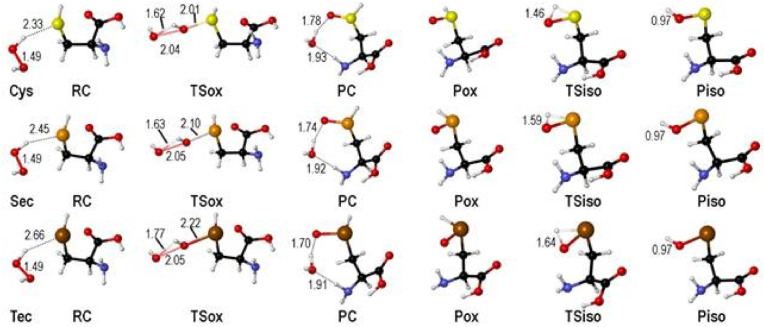
Stationary points along the stepwise oxidation mechanism of Cys/Sec/Tec by H_2_O_2_ (see Scheme 10). Relevant interatomic distances are shown in Å. Adapted from [38].

In all cases, the reaction goes through similar structures. When hydrogen peroxide approaches to the chalcogen nucleus, a reactant complex (RCox) is formed. The oxidation occurs crossing a transition state (TSox), where the O-O bond of hydrogen peroxide is breaking apart, while the O-X (X=S, Se, Te) bond is forming. A weakly bonded product complex (PCox) is formed with the cleaved water molecule coordinated to the chalcogenoxide group. The structure of the product (Pox) is almost unchanged after removal of the water molecule. The isomerization proceeds crossing a transition state (TSiso) connecting the chalcogenoxide Pox to the chalcogenenic acid Piso (Figure 1) [38].

As previously anticipated, the isomerization step is characterized by a high energy transition states, whose energy decreases when going from Cys, to Sec and to Tec (Table 3).

**Figure 2:**
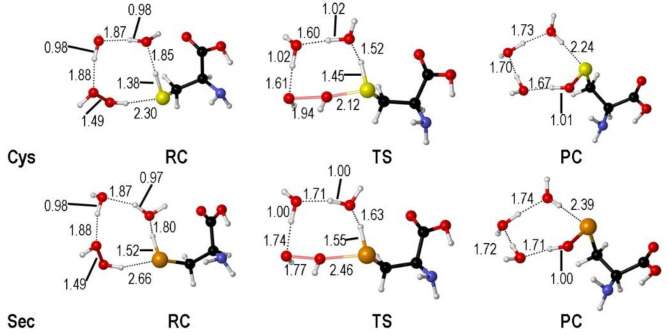
Stationary points along the water assisted oxidation mechanism of Cys/Sec by H_2_O_2_ (see Scheme 11). Relevant interatomic distances are shown in Å. Adaped from [38].

The transition states along the SAPE mechanism have been located only for Cys and Sec. In fact, this path is not found for Tec, in close analogy to the Tec oxidation path in GPx, where the formation of telluroxide is favored [39]. The TSs identified for Cys and Sec are rather similar; the X-H (X = S, Se) bond is elongated favoring the proton shuttling through the hydrogen bond network and the peroxide O-O bond is breaking apart (Figure 2).

When explicit water molecules are included in the model, the activation energy required for the oxidation of both free amino acids is lowered, as it was previously described for Cys, for which SAPE proved to be a valuable mechanism to reproducing experimentally detected activation energies [41]. 

Finally, when considering the anionic mechanism for Cys and Sec, the oxidation proceeds in a single step from chalcogenolates to deprotonated chalcogenenic acids, with a transition state connecting a reactant complex to a product complex that closely resembles those of the first pathway above described, i.e., oxidation of neutral residues. Notably, this mechanism shows the lowest activation energy for Cys and Sec oxidation, and this is justified by the enhanced nucleophilicity of deprotonated chalcogenols (Table 4) [38]. 

## 3. The Reduction of Hydroperoxides in Selenoproteins

Out of the various mechanisms of selenium-catalyzed H_2_O_2_ reduction, the oxidation of selenocysteine to the corresponding selenenic acid is the only one that has so far been detected in living organisms (see Scheme 11 and Figure 2). It here, however, does not occur with the free amino acid, but with a selenocysteynyl residue integrated into the amino acid chain of selenoproteins. Moreover, the particular environment of this selenocysteine residue opens up reaction possibilities that explain the unusually high catalytic efficiencies of these proteins (see below).

Among the selenoproteins, some members of the glutathione peroxidase family are directly in charge of balancing hydroperoxide challenge. The thioredoxin reductases indirectly contribute to this important task by providing reduced thioredoxin, the substrate of the majority of the peroxiredoxins. However, since hydroperoxides are not only toxic compounds that have to be eliminated from living systems, but also important signaling molecules [43,44], an over-optimization of hydroperoxide reduction may lead to unexpected disturbances of metabolic regulation. For instance, overexpression of glutathione peroxidase type 1 did not make mice more robust, but obese and insulin-resistant [45]. 

In selenoproteins, the reduction of H_2_O_2_ is regularly achieved by oxidation of a selenocysteynyl residue. This reaction takes place in many members of the glutathione peroxidase (GPx) family (for reviews see [46,47,48]) or in rare cases also in peroxiredoxins [49]. The common denominator of these enzymes is an oxidation of a selenocysteynyl residue to a selenenic acid and its reduction by a thiol. The formation of the selenenic acid could have never been proven by any analytical chemistry method, but is the only theoretical possibility, and was predicted by DFT calculations [50], and is chemically feasible [51]. It may sound risky to invoke an extremely unstable intermediate without analytical proof. Yet more recently, Masuda et al. [14] have provided NMR evidence that a cradle-protected selenocysteine can be oxidized to a selenenic acid and that the latter, like in glutathione peroxidases, readily reacts with thiols and less fast with NH-groups, while deselenylation is even less favoured. 

The reduction of the selenocysteynyl residues in the oxidized enzymes is always exerted by a thiol group. However, the specificities for both, the oxidizing hydroperoxide and the reducing thiol, differ substantially between these enzymes [48]. All glutathione peroxidases [46] and almost all peroxiredoxins [52] reduce H_2_O_2_ or other soluble hydroperoxides, while GPx4 appears to be specialized for the reduction of complex peroxidized lipids, even when firmly integrated into biomembranes. This role of GPx4 qualifies the enzyme (its cytosolic expression form) as a key regulator of ferroptosis. Many peroxiredoxins, however, also react with complex peroxidized lipids [52] and some appear to only reduce lipid hydroperoxides [53]. 

The reductive part of the catalytic cycles is even more diversified. GPx1, GPx2, GPx3 and GPx4 clearly prefer glutathione (GSH) as reducing thiol. However, GPx3 has been reported to be also reduced by thioredoxin, and GPx4 reacts with a non-physiological dithiols such as dithiothreitol and a realm of physiological protein thiols including cysteyl residues of GPx4 itself [47,54]. Polymerization of the mitochondrial GPx4 and co-polymerization with other cysteine-rich proteins yield a dead-end intermediate, which is an enzymatically inactive structural protein aggregate that is essential for male fertility in mammals [47,48]. GPx7 (and probably GPx8) prefers protein disulfide reductase as reducing substrate, thereby adopting an important role in oxidative protein folding. GPx7 [55] is also reduced by mortalin (also called GRP75, PBP74 or mtHSP70), thereby increasing the chaperone activity of the latter [56] GPx7 has also been reported to be an anti-oncogene [57]. The majority of cysteine-containing GPx homologs (see below) of bacteria, protozoa, plants and insects use thioredoxin or related redoxins as reductant [58], as do the 2-Cys-peroxiredoxins.

**Figure 3:**
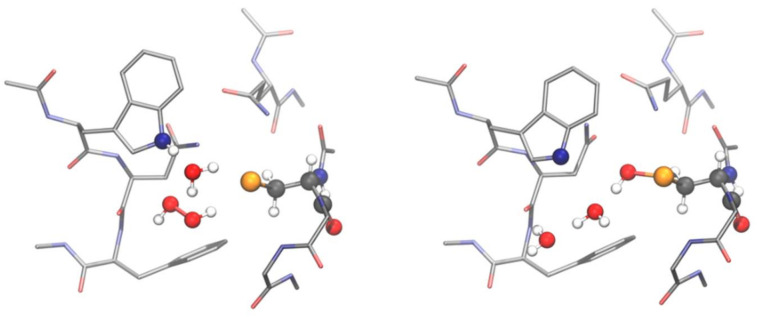
Stationary points along the PES of the GPx4 active site for the mechanism of oxidation of Sec by H_2_O_2_: zwitterionic intermediate (**left**) and selenenic acid (**right**). The fragments directly involved in the mechanism are shown in balls and sticks representation, while the remaining residues are shown in licorice representation and H atoms are omitted for clarity. Level of theory: SMD-B3LYP-D3(BJ)/6-311+G(d,p), cc-pVTZ//B3LYP-D3(BJ)/6-311G(d,p), cc-pVTZ [39].

Many attempts to explain the unusual kinetics and efficiencies of glutathione peroxidases have been published, but none of them reached any satisfactory solution, before the problem was investigated by DFT calculations [50]. When a model consisting of six conserved amino acid residues that constitute the active site in almost all glutathione peroxidases was calculated, the selenocysteine residue was seen to be dissociated (Figure 3 left). This finding was not a surprise, since selenocysteine has a low pK anyway (pK = 5.2). But surprisingly, also a cysteine (pK > 8), in this peculiar environment, proved to be dissociated. However, the proton leaving the selenocysteine or cysteine, respectively, remains bound in the active site. In case of GPx, it migrates water-mediated to the ring nitrogen of the strictly conserved tryptophan residue, which creates a zwitterionic structure (Figure 3 left). When an H_2_O_2_ molecule is added in a suitable orientation, the complex decays without any measurable activation energy. The selenium (or sulfur) makes a nucleophilic attack on one oxygen of the peroxide bond, while the dislocated proton, again mediated by water, attacks the second oxygen. By this dual attack, the peroxide bond is cleaved, and the selenenic (sulfenic) acid and a water molecule is formed (Figure 3 right). This mechanism, for the first time, complies with the unusual kinetic behaviour of glutathione peroxidases. An enzyme/H_2_O_2_ complex can never accumulate, since it reacts faster than it is built. This is in line with the lack of saturation kinetics (Michaelis constants and maximum velocities are infinite) [30,59]. The rate-limiting step in this scheme is a productive bimolecular collision of H_2_O_2_ with the zwitterionic form of the ground state enzyme, which may be considered to be very fast and, thus, complies with the unusually high rate constant for the reaction of the enzyme by H_2_O_2_ (ca 10^8^ M^−1^s^−1^ for bovine GPx1 [30]; more values are compiled in [60]).

As already briefly mentioned above, the selenocysteine in glutathione peroxidases can be replaced by cysteine without changing the basic mechanism of H_2_O_2_ reduction. If Cys homologues of the real selenoproteins are generated by site-directed mutagenesis, a dramatic decrease of specfic activity [28] and the rate constants for both, the oxidative and the reductive part of the catalytic cycle is observed [29]. Naturally, this substitution occurs in GPx5, GPx7 and Gpx8 of man and in the maiority of non-vertebrate glutathione peroxidase-type proteins. Despite the lack of selenocysteine these “CysGPxs” sometimes show very high reaction rates (up to 10^6^ M^−1^ s^−1^; [60]). Nevertheless, the CysGPxs are generally less efficient than their selenium-containing relatives. This observation complies with a recent DFT study with a simplified GPx model revealing that the energetics of the selenium-based catalysis are generally more favorable [61]. The catalytic superiority of the SecGPxs is in line with the enhanced reaction rates observed in the cystamine/selenocysteamine exchange reaction [62] and the largely increased activities when one or both sulfur atoms in the CPYC motif of glutaredoxin were exchanged by selenium [63]. Also in these cases, the higher nucleophilicity and polarizability of selenolate, when compared to thiolate, are discussed. Apart from the quantitative difference in the reaction of the chalcogens with H_2_O_2_, there is a qualitative difference in a downstream reaction of the GPx catalysis. When the natural selenium-containung GPx1 or GPx4 was incubated with H_2_O_2_ in absence of any reducing substrate, their selenenic acids reacted with an amide nitrogen of the protein backbone, while the artificial cysteine homologs became overoxidized to sulfinic and ultimately to sulfonic acids [50]. This difference reflects the tendency of selenium to stay in lower oxidation states than sulfur [27]. The Se-N bond formation in the natural glutathione peroxidases is readily reversed by GSH [50]. It is therefore considered as a a mechanism of self-protection that avoids irreversible destruction of the enzyme due to deselenylation.

The dual attack principle to cleave the peroxide bond, as first detected for glutathione peroxidases [50], was later extended to the peroxiredoxins [64]. Here the proton of the peroxidative cysteine residue migrates to the oxygen of a highly conserved and essential threonine [65]. This residue can be replaced by serine, but not by any residue lacking the OH group. Also proteins that are generally not classified as peroxidases, e.g., glyceraldehyde-3-phosphate dehydrogenase (GAPDH) or the bacterial hydroperoxide sensor OxyR, but react with H_2_O_2_ much faster than fully dissociated low molecular mass thiols (*k* > 50 M^−1^ s^−1^) make use of the dual attack principle [64,66,67]. It appears promising to investigate further protein families, which have been reported to have super-reactive cysteine residues, for this possible mechanism.

## 4. The Reduction of Hydroperoxides by GPx Mimics: The Case of Ebselen

**Scheme 13:**
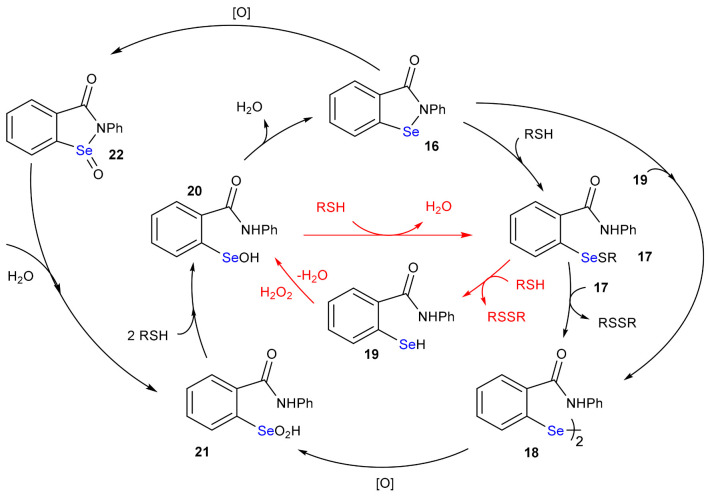
GPx-like mechanism of ebselen. The steps of the GPx-like path are highlighted in red.

The presence of selenium in enzymes involved in the endogenous enzymatic defense against harmful oxidants soon prompted for the design and synthesis of bioinspired molecules with antioxidant potential, i.e., capable of efficiently reduce peroxides [68,69,70]. Particularly, in the last fifty years, numerous compounds mimicking GPx activity have been prepared and tested in vitro and in vivo [71]. From a chemical point of view, they can be grouped into three classes, i.e., diaryldiselenides, selenenylamides and alkylselenides [72]. While these last compounds have been less investigated, likely for their adverse effects, diphenyl diselenide and its derivatives have been thoroughly studied [13,24]. But the most interesting mimics undoubtedly belong to the group of selenenylamides and among these, ebselen (**16**, 2-phenyl-1,2-benzoselenazol-3(2H)-one) [73,74] has been employed in clinical trials, due to its low toxicity. The GPx like mechanism of ebselen (Scheme 13) was studied in detail by Antony and Bayse [40]. In water, the energetically preferred mechanism consists in the subsequent reaction with two equivalents of thiol, **16**→**17** (Gibbs free activation energy: 8.4 kcal mol^−1^ and Gibbs free reaction energy: −14.4 kcal mol^−1^) and **17**→**19** (Gibbs free activation energy: 31.7 kcal mol^−1^ and Gibbs free reaction energy: +11.8 kcal mol^−1^) with the formation of the selenol **19** which reacts with the peroxide (**19**→**20**; activation energy: 12.8 kcal mol^−1^ and reaction energy: −68.0 kcal mol^−1^) [40]. The disproportionation step **17**→**18** was excluded, also because it implies a bimolecular reaction of two equivalents of catalyst, and thus also the oxidation **18**→**21** can be ruled out. Compound **21** can be formed by hydrolysis of the ebselen selenoxide **22**, which is obtained oxidizing **16**. However, although **16** reacts with H_2_O_2_, its reaction with thiols is competitive and is the preferred pathway under most conditions [40]. 

The therapeutic use of ebselen is continuously debated. Clearly, its use as antioxidant GPx mimic has failed. In fact, in physiological environment ebselen is mainly bound to the cysteine thiols of serum albumin. Based on the energy values above reported, the reduction of the Se-S bond to regenerate the selenol form present in **19** has a high activation energy and is an endergonic process. Conversely, the thiol exchange reaction, which implies a nucleophilic attack of a thiolate at selenium in **17**, is thermodynamically favored and has paved the route to a completely different potential use of ebselen, i.e., enzymatic inhibitor. The mechanisms of these nucleophilic substitutions have been tackled in detail by several authors [25,75,76,77,78]. Among the examples reported in literature, it is worth to mention the antidepressant lithium-like potential of ebselen as inositol monophosphatase inhibitor [79,80], as disruptor of protein Zn fingers [81,82], and, more recently, ebselen has been repurposed as SARS Cov 2 M^pro^ inhibitor [83,84,85,86], justifying its antiviral activity measured in vitro. 

## 5. Deselenylation Paths

**Scheme 14:**
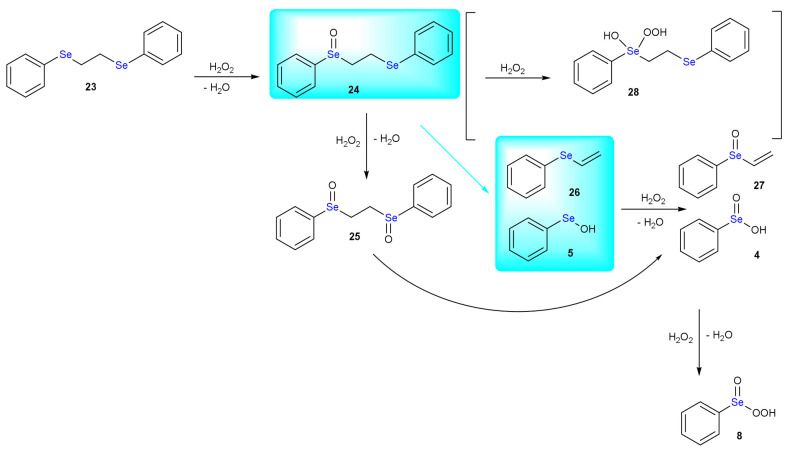
Mechanism of the oxidation of PhSeEtSePh by H_2_O_2_. The direct selenoxide elimination is highlighted in cyan.

The thermal instability of organic sulfoxides was already known in 1875 [87]. In the Sixties, the pyrolysis of unsymmetrical dialkyl sulfoxides was systematically investigated [88]. Upon heating at temperatures close to 200 °C, the sulfoxides decompose to afford olefinic products. Particularly, the rate of alkene formation increases when both or at least one substituent is a secondary alkyl group rather than primary. The products are explained invoking an intramolecular elimination involving the sulfenate group and one available β hydrogen. Selenoxides undergo analogous reaction more rapidly, which occurs at much lower temperatures (less than 40 °C). The syn elimination requires the coplanarity of the C-H and Se-C bonds in the transition state. The selenoxide elimination is favored by the presence of an adjacent carbonyl group and it is considered a general method for the preparation of α,β-unsaturated carbonyl compounds. In [17], it has been shown that oxidation of 1,2-bis(phenylselanyl)ethane (PhSeEtSePh, **23**) by H_2_O_2_ leads to an olefin product with loss of one selenium group. In fact, as shown in Scheme 14, upon addition of one equivalent of peroxide the selenoxide **24** forms (activation energy of 17.4 kcal mol^−1^ and reaction energy of −35.8 kcal mol^−1^). Very similar energy values are computed for the oxidation of the second selenium center, i.e., +17.0 and −36.7 kcal mol^−1^, leading to the formation of the diselenoxide **25**. Interestingly, β elimination occurs from the diselenoxide **25**, leading to 1-(vinylseleninyl)benzene **27** and benzene seleninic acid **4**, requiring an activation energy of 19.5 kcal mol^−1^ and being slightly endergonic (+0.6 kcal mol^−1^). But if the elimination occurs from the selenoxide **24** leading to the formation of the alkene **26** and benzene selenenic acid **6**, the activation energy is 21.2 kcal mol^−1^ and the reaction energy becomes −3.0 kcal mol^−1^. The product is then oxidized to 1-(vinylseleninyl)benzene **27** with an activation energy of 19.3 kcal mol^−1^ and energy release of 33.1 kcal mol^−1^. Benzene selenenic acid **6** is then stepwise oxidized to benzene seleninic acid **4** and benzene peroxyseleninic acid **8** (activation energies of 12.6 kcal mol^−1^ and 24.8 kcal mol^−1^ and reaction energies of −49.2 kcal mol^−1^ and −5.5 kcal mol^−1^, respectively). Importantly, the formation of the benzene peroxyseleninic acid **8** triggers an autocatalytic mechanism and the oxidation of the diselenide **23** by this latter has a barrier of 8.1 kcal mol^−1^ and a reaction energy of −30.3 kcal mol^−1^ leading to benzene seleninic acid **4** and selenoxide **24**. Notably, the formation of the hydroxy perhydroxy selenane derivative **28** is energetically not favored.

In this example, the formation of the alkene is unwanted, but the convenience of the elimination process can be fully exploited to prepare peculiar olefins and other organic compounds of interest. A very recent combined experimental and computational study describe a selenium derivative of fluoxetine which is subjected to oxidation-elimination and represents a green chemistry approach to the trans-selective production of cynnamylamines [89]. Notably, starting from a monoselenide, the olefin product does not contain selenium.

**Scheme 15:**
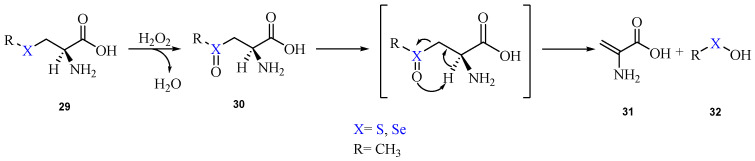
Oxidation of methyl(seleno)cysteine by H_2_O_2_ and subsequent elimination, leading to the DHA formation.

Recently, with the aim of gaining insight into this class of reactions, the oxidation and β-elimination pathways of methyl(seleno)cysteine have been thoroughly investigated at ZORA-BLYP-D3(BJ)/TZ2P level of theory [90]. The mechanism is shown in Scheme 15.

**Figure 4:**
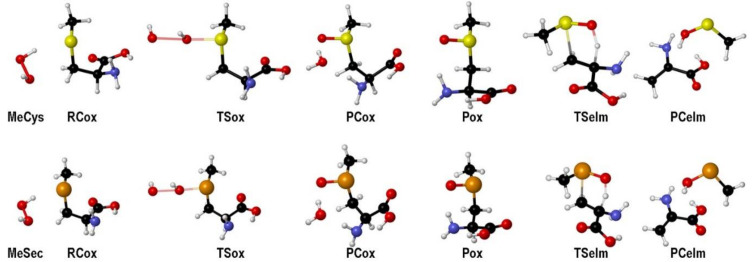
Stationary points along the oxidation mechanism of MeCys/MeSec by H_2_O_2_ (see Scheme 14) and subsequent elimination. Adapted from [90].

For both MeCys and MeSec, the oxidation proceeds through a transition state connecting a weakly bonded reactant complex (RCox) to a weakly bonded product complex (PCox). The elimination reaction is an intramolecular process (E_i_), characterized by β-proton abstraction, leading to a product complex for the elimination (PCelm) and leads to the formation of DHA and a chalcogenenic acid (Figure 4).

The Gibbs free activation and reaction energies are shown in Table 5.

**Figure 5:**
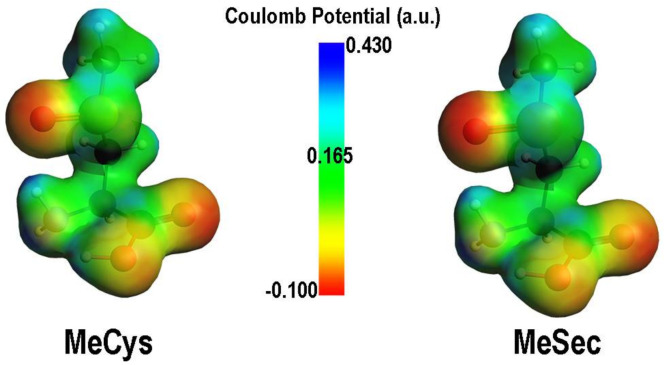
Comparison between MEPs of the oxidized forms of MeCys and MeSec. Adapted from [90].

The elimination is rate determining in both cases, and it is much more favoured in presence of selenium. Moreover, selenoxides are far less stabilized with respect to sulfoxides, because of a more dipolar character of the bond and weaker π-character of X=O double bond when Se is involved, because of an elongated X=O bond (Figure 5). The products and PC for the elimination are also more stabilized when selenium is involved. All these results explain the facile route to DHA formation in presence of selenium [90].

The selenoxide elimination might in principle occur also in biological environment upon oxidation of the selenium-based residues. Deselenylation of unproperly stored selenoproteins was repeatedly reported [91,92]. In 2010, Cho et al. speculated that deselenylation of GPx1 due to H_2_O_2_ exposure may even adopt a physiological role [93]. Its Sec residues were transformed to dehydroalanine (DHA) by treatment of isolated GPx1 with 1 mM H_2_O_2_ for one hour. This transformation was shown to be irreversible as opposed to the overoxidation of sulfinic acid in peroxiredoxin II. However, the physiological steady –state concentrations of H_2_O_2_ are estimated to range from 10^−9^–10^−7^ M. They may locally be a bit higher, but they will never reach 1 mM for a whole hour. Morover, Cho et al. detected the dehydroalanine after tryptic digestion of the protein, which dramatically increases the deselenylation [50]. In a commercial, which means aged, sample of bovine red blood cell GPx1, Orian et al. could hardly detect any dehydroalanine. Oxidation of the enzyme by 5 μM H_2_O_2_ in absence of any reducing substrate yielded an oxidized enzyme that contains a Se-N that is readily cleaved by glutathione and, thus, the inhibition associated with this bypass is reversible. Moreover, an almost 100% conversion of the Sec residues to dehydroalanine is found after tryptic digestion [50]. This transformation could be inhibited by a thiol to mimic the canonical catalytic cycle of GPxs. In short, deselenylation of the Sec residue in GPxs appears not to be of any physiological relevance.

## 6. Conclusions

We have revisited the mechanistic details of organoselenides oxidation by hydroperoxides, mostly hydrogen peroxide. In synthetic organic chemistry, this process is largely exploited to lead selenium to high oxidation states (perseleninic acid) to be employed as efficient oxidizing agent. In biology, selenium is in the core of enzymes deputed to consuming excess of hydroperoxides, which are harmful for the cell, or to exploiting the chemical reaction of hydroperoxide reduction for signalling purpose. The same elementary reaction, which can be modelled as a nucleophilic attack of the selenolate anion to the peroxide O-O bond, is fundamental in chemistry as well as in biology. In chemistry, acidity and nucleophilic properties of selenium and its capacity of behaving like and effective oxygen transfer agent reaching high oxidation states fully account for its catalytic and autocatalytic behaviour. In enzymatic pockets, selenium properties are advantageously finely tuned: nucleophilicity is boosted by the capacity of surrounding residues to accept and donate to the substrate the selenol proton and its redox properties are modulated so that selenium reaches the selenenic state and its overoxidation is prevented thus minimizing the risk of irreversible selenoxide elimination leading to DHA. These properties make selenium a unique catalytic center. Based on the experimental observations and theoretical rationalization, the difficulties in preparing efficient molecular selenium-based antioxidants are clear. The reactivity of these systems cannot be regulated like in the protein environment, where the surrounding residues take part into the chemical mechanisms. On the other hand, this paves the route to a different modern vision of selenium applications in health sciences, based on its prooxidant properties rather than on its antioxidant GPx mimic potential. 

## Figures and Tables

**Table 1:** Energy barriers for the oxidation of phenylchalcogenols by H_2_O_2_ (Scheme 9A). Level of theory: ZORA-OLYP/TZ2P. Data taken from [26].

Elementary Steps	S	Se	Te
**I**	27.1	24.0	16.5
**iso**	38.8	31.0	31.5
**II**	19.3	16.8	9.4
**III**	26.2	32.3	29.8

**Table 2:** Energy barriers for the oxidation of phenylchalcogenolates by H_2_O_2_ (Scheme 9B). Values in parentheses were computed in water. Level of theory: (COSMO)-ZORA-OLYP/TZ2P. Data taken from [26].

Elementary Steps	S	Se	Te
**I**	12.3 (7.4)	10.2 (5.5)	8.2 (3.7)
**II**	11.2 (8.4)	12.0 (9.4)	12.0 (8.9)
**III**	11.2 (10.0)	16.5 (16.2)	16.8 (16.4)

**Table 3:** Electronic energies (kcal mol^−1^) relative to free reactants for the oxidation of free protonated (Scheme 11) and deprotonated amino acids (Scheme 13). Level of theory: ZORA-BLYP-D3(BJ)/TZ2P. ^a^ Data taken from [38].

	RC_OX_	TS_OX_	PC_OX_	P_OX_	ΔE^#^_OX_^a^	TS_iso_	P_iso_	ΔE^#^_iso_^b^
**Cys**	−6.01	17.84	−47.88	−38.84	23.85	39.80	−49.83	39.80
**Sec**	−6.02	14.06	−40.04	−29.64	20.08	30.77	−55.42	30.77
**Tec**	−6.04	6.25	−48.51	−37.72	12.29	32.09	−65.75	32.09
**Cys^−^**	−19.86	−13.01	−65.14	−50.17	6.85	-	-	-
**Sec^−^**	−18.70	−13.51	−59.92	−44.08	5.19	-	-	-

^a^ Activation energies relative to RC_OX_. ^b^ TS and activation energies relative to P_OX_.

**Table 4:** Electronic energies (kcal mol^−1^) relative to free reactants for the oxidation of free amino acids (Scheme 12) along the SAPE pathway. Level of theory: ZORA-BLYP-D3(BJ)/TZ2P. ^a^ Data taken from [38].

	RC_OX_	TS_OX_	PC_OX_	P_OX_	ΔE^#^_OX_^a^
**Cys**	−22.92	−12.65	−80.81	−49.83	10.27
**Sec**	−22.43	−15.67	−84.88	−55.42	6.77

^a^ Activation energies relative to RC_OX_.

**Table 5:** Gibbs free energies (∆G, kcal/mol) relative to the free reactants for the oxidation and sequential β-elimination of MeCys and MeSec in water. Activation and reaction Gibbs free energies are relative to RC_ox_ for the oxidative step and to P_ox_ for the elimination. Level of theory: COSMO-ZORA-BLYP-D3(BJ)/TZ2P. Data taken from [90].

	RC_ox_	TS_ox_	ΔG^‡^_ox_	PC_ox_	P_ox_	TS_elm_	ΔG^‡^_elm_	PC_elm_	P_elm_	ΔG_elm_
**MeCys**	2.65	10.24	7.59	−47.48	−49.25	−30.94	18.31	−42.52	−48.17	1.08
**MeSec**	3.80	6.37	2.57	−41.24	−41.87	−31.64	10.23	−45.99	−51.58	−9.71

## Abbreviations

BP86	GGA functional with Becke exchange and Perdew correlation
B3PW91	Hybrid functional with Becke exchange and Perdew Wang correlation
COSMO	Conductor-like Screening Model for solvation
Cys	Cysteine
DFT	Density Functional Theory
DHA	Dehydroalanine
GAPDH	Glyceraldehyde 3-phosphate dehydrogenase
GGA	Generalized Gradient Approximation
GPx	Glutathione Peroxidase
GTO	Gaussian Type Orbital
IMPase	Inositol MonoPhosphatase
OLYP	GGA functional with OPTX exchange and LYP correlation
OPBE	GGA functional with OPTX exchange and PBEc correlation
D3(BJ)	Dispersion correction (D3) by Grimme with damping function
HOMO	Highest Occupied Molecular Orbital
LUMO	Lowest Unoccupied Molecular Orbital
MeCys	Methyl Cysteine
MEP	Molecular Electrostatic Potential
MPn	Møller Plesset perturbative method of order n
MeSec	Methyl Selenocysteine
NMR	Nuclear Magnetic Resonance
PC	Product Complex
PCM	Polarizable Continuum Model
PES	Potential Energy Surface
QCISD(T)	Quadratic Configuration Interaction with Single Double (Triple) excitations
QM	Quantum Mechanics
QZ4P	uncontracted set of Slater-type orbitals of quadruple-ζ quality, augmented with four sets of polarization functions per atom
RC	Reactant Complex
SAPE	Solvent Assisted Proton Exchange
Sec	Selenocysteine
S-GPx	Sulfur mutant of glutathione peroxidase
SMD	Solvation Model based on Density
STO	Slater Type Orbital
Tec	Tellurocysteine
Te-GPx	Tellurium mutant of glutathione peroxidase
TS	Transition State
TZP	uncontracted set of Slater-type orbitals of triple-ζ quality, augmented with one set of polarization functions per atom
TZ2P	uncontracted set of Slater-type orbitals of triple-ζ quality, augmented with two sets of polarization functions per atom
XC	Exchange- Correlation
ZORA	Zeroth Order Regular Approximation
